# The Rheumatic and Gouty Diathesis as Manifested in Diseases of the Peridental Membrane

**Published:** 1891-10

**Authors:** John S. Marshall

**Affiliations:** No. 7 Jackson Street, Chicago


### THE RHEUMATIC AND GOUTY DIATHESIS AS MANIFESTED IN DISEASES OF THE PERIDENTAL MEMBRANE.[1]

BY JOHN S. MARSHALL, M.D.[2]

In nearly all our text-books on dental surgery and dental pathology, mention is made of the fact that rheumatic and gouty individuals sometimes suffer from irritation of the peridental membrane, causing the teeth to become more or less sore and loosened in their sockets; supposedly the results of the peculiar diathesis of the system, but further than this no light is thrown upon the subject.

Clinical and post-mortem experience teaches that the *materies morbi* of these diseases has a predilection for the fibrous structures of the body, especially the synovial membranes, the aponeuroses of muscles, the dura-mater, the cardiac tissues, periosteal and peridental membranes. The one most commonly affected is the synovial membrane, resulting in inflammatory conditions of the joints.

It not uncommonly happens, however, that there is associated with the inflammatory phenomena of the joints, enlargement of the long bones and nodular formations in other localities; and in the cementum and pericementum, conditions which are somewhat analogous.

The predisposing and exciting causes of certain irritative conditions of the pericementum seem to have their origin in the same conditions which bring about the phenomena of gout and rheumatism, and they have also proved by experience to be amenable, in many cases, to the same specific treatment adopted in these dis-

---

[1] Read before the Oral Surgical Section of the American Medical Association, Washington, D.C., 1891.

[2] Professor of Oral Surgery, University Dental College, Chicago.

eases. This last fact has led me to the belief that the rheumatic poison is largely accountable for many of the diseased conditions of the pericementum, and has induced me for several years to make close and careful observation in relation to the prevalence of irritation of the pericementum in gouty and rheumatic individuals.

This belief has been further strengthened by finding, upon analyzing the urine of a number of persons suffering from peridental irritation of this character, that there was a considerable excess of uric acid in every case. The saliva, also, in many cases gives a decided acid reaction.

The peridental membrane seems to be very susceptible to the irritating effects of an acid condition of the blood, whether from an excess of lactic or uric acids retained in the system or from the ingestion of such acids as are found in sour wines and malt liquors.

The habitual use of sour wines and malt liquors often results in peridental irritation of a marked character in individuals who have never developed symptoms of a rheumatic condition; while on the other hand the same irritative conditions are found in total abstainers; but these, it would seem, are due to rheumatic phenomena, and are often the forerunner of an approaching attack of acute articular inflammation.

Congestion and thickening of the peridental membrane and temporary loosening of the teeth, accompanied with dull, gnawing pains and more or less soreness, are a common occurrence in rheumatism and gout. At times this condition is the first definite symptom to be manifested of an approaching attack of acute articular inflammation, while in others it does not make its appearance until after the first acute symptoms have subsided, and occasionally it is the only manifestation of this peculiar diathesis.

Chilling the surface of the body, or, in other words, taking cold, is usually the exciting cause of an attack, while an injudicious diet greatly aggravates it.

The presence of concretions upon the roots of the teeth is the most common cause of inflammation of the pericementum, and it has been generally supposed that these concretions were formed from the saliva.

It is my purpose to call attention to what seems to me to be a more rational explanation of the formation of these deposits in locations where the saliva could not, or could with great difficulty, reach. The saliva contains, as waste products, a certain amount of phosphate and carbonate of calcium which has been rendered soluble by the action of carbonic acid. A certain amount of ammonia is given off from the lungs as a product of decomposition of tissue; while fermentation of alimentary *débris* lodged about the teeth also furnishes an additional amount of ammonia. This ammonia, coming in contact with the saliva in the mouth, unites with a portion of the carbonic acid to form carbonate of ammonia, thus liberating a portion of the calcium, which is thrown down in the form of a precipitate and lodges upon the exposed surfaces of the teeth. But when deposits occur at remote points from the gum-margin, it does not seem possible that this is the correct explanation of their presence in these locations. The law of gravity carries all bodies that are heavier than the medium in which they are suspended downward; consequently we must look for some other explanation for the presence of the deposits on the roots of the superior teeth.

Capillary attraction may possibly account for their location, but this seems hardly probable, for it presupposes the presence of a pocket, or a separation of the pericementum from the cementum, while the amount of saliva entering such an existing *cul-de-sac* would be extremely small, and not likely to be changed at sufficiently frequent intervals to account for the rapid accumulation which takes place in some of these cases. We are forced, therefore, to the supposition that they are deposited from some other source, and under an entirely different group of circumstances.

It has been suggested by Dr. Ingersoll, of Keokuk, Iowa, that these concretions were a direct deposition from the *liquor sanguinis* which bathes the roots of the teeth during the suppurative stage of the inflammatory process. This may be the correct solution. It is true that calcareous material is sometimes deposited from pus, in proof of which might be mentioned the fact that the roots of teeth penetrating the antrum of Highmore and foreign bodies located in this sinus during suppurative inflammation have been found when removed to be covered with calcareous deposits, but that this is a common occurrence in suppurative conditions in any locality admits of serious question. I am of the opinion, however, that the deposition of the concretions upon the roots of the teeth in those localities not readily reached by the saliva, or in which the presence of the saliva would be an impossibility, are due to the causes which produce the chalky formations found in the joints and fibrous tissues of gouty and rheumatic individuals.

The thought has occurred to me, though I have not had time to demonstrate it positively, that the concretions found upon the roots of the teeth in the locations just named were masses of urate of soda with phosphate and carbonate of calcium, and that they are deposited directly from the secretions, as is often the case in rheumatic arthritis.

Furthermore, it would seem that these concretions were the cause of the inflammatory condition, rather than the result of it; in proof of this let me state that clinical observation teaches that suppuration often occurs about the roots of the teeth at remote points from the gum-margin, and which have no outlet until the pericementum is dissected from the roots of the teeth by the accumulation of the pus. I have seen cases in the lower jaw in which an abscess had been formed upon the roots of living teeth, between the neck and the apex, and in which the attachment of the gum at the neck of the tooth was intact, and the pus did not escape until nature had perforated the soft tissue, or relief was given by the use of the knife. In such cases I have never failed to find concretions upon the root at the point of suppuration; this could not possibly have been deposited from the saliva; and it is fair to presume that the deposits upon the root was the source of irritation that produced the abscess, rather than that the inflammatory condition was produced by some remote cause and the formation of the deposit the result of the inflammation.

Dental exostosis, or hypertrophy of cement tissue, is an occasional occurrence in individuals of a gouty or rheumatic diathesis. It is most commonly found in chronic cases of long standing and is often associated with enlargement of the joints.

The causes are in all probability the same as those which produce the enlarged joints. Chronic irritation of the periosteum tends to hypertrophy of bone; while the same condition of irritation in the pericementum produces hypertrophy of the cementum. The history of these cases is usually one of chronic irritability of the pericementum, with periodical attacks of soreness and looseness of the teeth, while the cause of each attack is generally referred to some recent exposure in which the individual has taken cold. The acid reaction of the saliva and the urine at these times gives evidence of an acid condition of the blood. This would seem to indicate the cause of the irritation and suggest the line of treatment to control the immediate symptoms and the prophylaxis of the future.

*Phagedenic pericementitis* is sometimes directly traceable to a rheumatic condition of the system or the uric acid diathesis. In several cases in which I have analyzed the urine, uric acid was found largely in excess of the normal quantity.

In all of the cases which I class as rheumatic, concretions are found upon the roots of the teeth, and many times in locations which preclude the possibility of a salivary origin.

Under a restricted diet in which meats, wine, and malt liquors are cut off there is soon a marked diminution of the quantity of uric acid excreted, and an equally marked improvement takes place in the symptoms manifested in the oral cavity, which cannot be accounted for by the removal of the concretions and local treatment alone. In one case, which has been under observation for four years, the periodical aggravation of the oral symptoms is a sure indication of the presence of an excessive amount of uric acid in the urine, and as soon as this condition is corrected the inflammatory conditions of the pericementum are greatly relieved. Local treatment is necessary, but this alone is not sufficient; we must strike deeper and correct the morbid condition of the system if we hope to effect a cure.

Other cases might be cited that would seem to further substantiate the opinions already expressed, but time will not permit. I trust, however, the matter has been so presented as to stimulate further investigation into this subject, and I believe if your observations are carefully made you will agree with me that the facts and opinions presented warrant the supposition that gout and rheumatism are important factors in the production of a considerable variety of irritative conditions of the peridental membrane.

No. 7 Jackson Street, Chicago.

